# Light-matter decoupling and **A**^2^ term detection in superconducting circuits

**DOI:** 10.1038/srep16055

**Published:** 2015-11-04

**Authors:** J. J. García-Ripoll, B. Peropadre, S. De Liberato

**Affiliations:** 1 Instituto de Física Fundamental IFF-CSIC, Calle Serrano 113b, Madrid E-28006, Spain; 2 Department of Chemistry and Chemical Biology, Harvard University, Cambridge MA, 02138; 3 School of Physics and Astronomy, University of Southampton, Southampton, SO17 1BJ, United Kingdom

## Abstract

The spontaneous and stimulated emission of a superconducting qubit in the presence of propagating microwaves originates from an effective light-matter interaction that, similarly to the case of the atomic case, can contain a diamagnetic term proportional to the square vector potential **A**^2^. In the present work we prove that an increase in the strength of the diamagnetic term leads to an effective decoupling of the qubit from the electromagnetic field, and that this effect is observable at any range of qubit-photon coupling. To measure this effect we propose to use a transmon suspended over a transmission line, where the relative strength of the **A**^2^ term is controlled by the qubit-line separation. We show that the spontaneous emission rate of the suspended transmon onto the line can, at short distances, increase with such a separation, instead of decreasing.

When the vacuum Rabi frequency, Ω, of an electromagnetic mode is much smaller than the bare frequency of the excitation to whom it couples, *ω*, the simple Jaynes-Cumming or Tavis-Cumming models capture the main features of light-matter interaction and cavity QED^1^,^2^. However, already for a normalised coupling 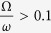, the Rotating Wave Approximation (RWA) that justifies those solvable models fails^3^. In this ultrastrong coupling (USC) regime, light-matter interaction must be described beyond RWA, using the Rabi^4^ and Hopfield-Bogoliubov^5^ models, that correctly describe the ground state squeezing and asymmetric splitting^6^,^7^,^8^,^9^. The USC regime, observed for the first time only few years ago^10^, has now been achieved in many solid-state cavity quantum electrodynamics setups^11^,^12^,^13^,^14^,^15^,^16^,^17^, with an actual coupling record of 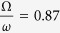
18. When the normalised coupling becomes of the order one, also the aforementioned non-RWA models fail. In this regime, named deep strong coupling (DSC)^19^, the localised dipolar interaction dominates and a real-space description with many excited photonic modes becomes essential.

Our understanding of such a deep non-perturbative regime is still incomplete^20^,^21^,^22^,^23^,^24^. A first, recent counter-intuitive result is that light and matter eventually decouple in the DSC regime: the spontaneous emission rate of the system dramatically decreases, instead of increasing, with the coupling strength^25^. This decoupling is associated with the diamagnetic term **A**^2^, that expels modes away from the emitter. Still, the decoupling has been rigorously proved only for linear systems —a perfect planar metallic cavity coupled to a 2D sheet of dipoles—, and the link of the decoupling effect with the **A**^2^ term remains a hypothesis. Indeed, without diamagnetic term, the model in ref. 25 becomes unstable and undergoes a superradiant phase transition^26^,^27^,^28^, impeaching a comparison of DSC physics with and without **A**^2^.

In circuit QED it has been shown that a microscopic treatment of the light-matter coupling between a superconducting waveguide and a qubit gives rise to a diamagnetic term, analogous to the **A**^2^ term of the minimal coupling Hamiltonian^29^. Still, contrary to the usual minimal coupling case, the relative strength of the dipolar and diamagnetic terms is not fixed by the Thomas-Reiche-Kuhn sum rule. Circuit QED setups thus give us the opportunity to study the intrinsic behaviour of the light-matter coupling while freely tuning the intensity of the diamagnetic term, and without risk to encounter unstable regimes^30^.

The first major result of this paper is to prove that the decoupling effect happens already at the *single dipole* limit, and moreover, in circuit QED systems, it can be observed *at all levels of dipolar interaction strength*, that is without the need to be in the USC/DSC regimes. We arrive at this result by studying the nonlinear interaction between a two-level system and a one-dimensional waveguide, modeled by the Ohmic spin boson model^31^,^32^, and proving that the spontaneous emission rate of the two-level system decreases with the intensity of the **A**^2^ term. While this model, with its continuous spectrum, is more complex that the discrete mode approach usually employed in circuit and cavity QED, it is necessary to study the decoupling effect in its generality. In fact it allows us to factor out resonance effects and study the intrinsic impact of the diamagnetic term on the light-matter coupling.

The second major result is to propose an experiment, realisable with present day technology, to measure such an intensity. Actual experiments cannot determine the weight of the dipolar and diamagnetic terms individually, because the effective light-matter interaction model remains Ohmic and described by a single parameter, the spontaneous emission rate. In order to solve this problem, we consider a superconducting circuit consisting of a transmon qubit^33^,^34^ suspended on top of a microwave guide [cf. Fig. 1]. This setup, which profits from the ever improving coherence properties^35^,^36^ and strong interaction^11^,^37^,^38^ of superconducting circuits, was introduced in ref. 39 as an ultrasensitive scanning probe to locally analyze complex quantum simulators^40^. We prove that the same setup allows to control the relative strength of the diamagnetic term through the separation between the qubit and photon planes. The striking consequence of this control is that the coupling between the qubit and the line first increases and then decreases as the qubit is moved towards the line. This non-monotonic behavior is observable with *existing* transmon technology and does not need of USC/DSC regimes, opening the door to accurately calibrating the effective models that describe superconducting circuit quantum optics.

## Results

### General decoupling result

Let us start by discussing the main results in a formal spin-boson model. Using the qubit gap *ω*_0_ as unit of energy, the model reads


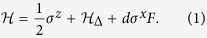


The photonic Hamiltonian, 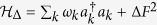, describes the free field in the waveguide, including the quadratic diamagnetic term weighted by the parameter Δ. The field operator 
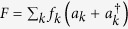
 depends only on the normal modes of the waveguide 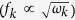, and it describes the field that interacts with the qubit through the dipole *d*. The exact physical interpretation of such a field depends on the specific circuit we are modelling, and in particular on what dynamical variable of the field the qubit couples to. In the following we will specifically consider two types of quibits: charge and flux ones, that couple capacitively and inductively with the waveguide. When referring to a specific kind of coupling, we will use the label *F*^(*cq*)^ for the charge qubit and *F*^(*fq*)^ or the flux one.

The strength of the light-matter interaction in the spin-boson model is determined by the spectral function 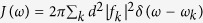. When Δ = 0 we recover the usual spin-boson model, characterized by the Ohmic spectral function^31^,^32^ *J*(*ω*; Δ = 0) = *d*^2^ × 2*παω*^1^, where *α* contains the details of *f*_*k*_. In the Markovian limit, *J*(*ω*_*qubit*_) = *J*(1) = 2*πα* gives the spontaneous emission rate of the qubit onto the transmission line, provided we are outside the localization or strongly correlated regimes, 2*πα* ≪ 1.

When Δ ≠ 0, we can recover again the usual spin-boson model by diagonalising the photonic Hamiltonian 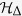, and rewriting the Hamiltonian [See Sect. IV A] in terms of its Δ-dependent normal modes,


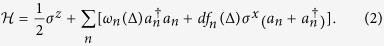


The mode frequencies and couplings^41^ now depend on Δ and have to be analyzed for each physical system. Our first result is that for a superconducting qubit the new spectral function remains Ohmic, so that the qualitative dynamics of the qubit with and without the diamagnetic term is the same. The second result is that the diamagnetic term leads to a reduction of the interaction strength *α*(Δ), and thus proves the generality of the decoupling effect studied in^25^. Moreover, we show how this coupling strength reduction could be potentially measured.

In Fig. 2(a) we show the outcome of our numerical simulations for a capacitive coupling, *F*^(*cq*)^. As shown in the plot, the model is Ohmic, but the slope is changed, decreasing with increasing Δ. This means that the parameter *α* measuring the coupling strength rapidly decreases with Δ. This is observed for both *F*^(*cq*)^ and *F*^(*fq*)^, as shown in Fig. 2(b). In particular, the capacitive coupling can be fit to 2*πα*^(*cq*)^ = (1 + 6.77Δ)^−2.57^, undistinguishable from the actual plot in Fig. 2(b).

### Decoupling of a transmon

So far we have been working with a dimensionless rewrite of the spin-boson model, where the microscopic parameters were abstracted into the dipole moment of the qubit *d* and the weight of the quadratic term Δ. In actual physical systems, both *d* and Δ depend on similar physical parameters and, even if *α*(Δ) decreases, the product *J*(*ω*) ∝ *d*^2^*α*(Δ) might not.

**Figure 1:**
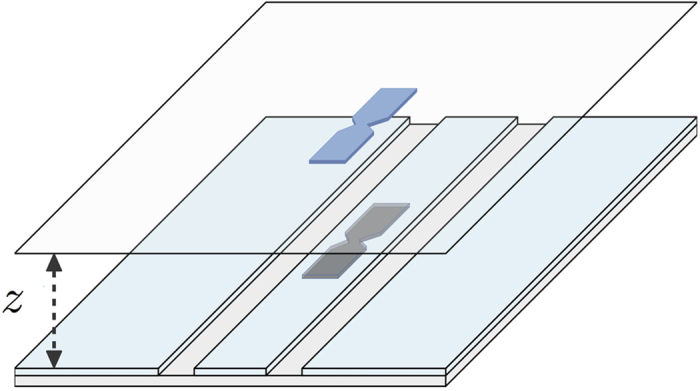
The spontaneous emission of a superconducting qubit, such as a transmon, suspended on top of a transmission line depends non-monotonically on the separation from the line, *z*, because of the influence of the A^2^ term.

**Figure 2:**
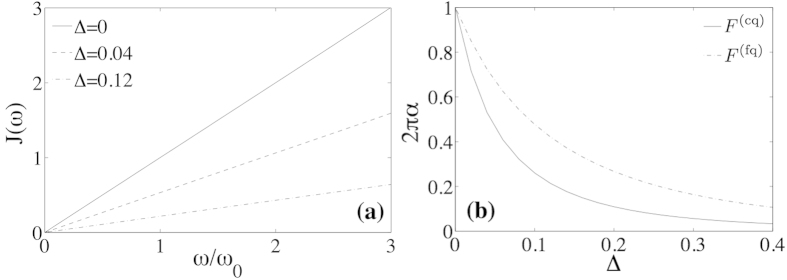
Light-matter decoupling in presence of the A^2^ term (Δ ≠ 0). Panel (**a**): spectral function for various values of Δ in a model with *F*^(*cq*)^ coupling. Panel (**b**): value of the coefficient *α* in a fit to *J*(*ω*; Δ) = 2*πα*(Δ)*ω*^1^ for the couplings *F*^(*cq*)^ and *F*^(*fq*)^. In both simulations *L* = 10*λ*_0_, and *v* = 1.

**Figure 3:**
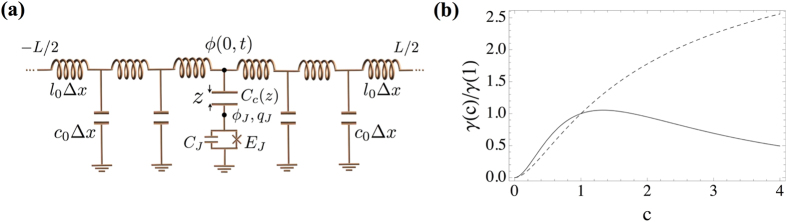
(**a**) Equivalent circuit of a suspended transmon capacitively coupled to an open transmission line, via a spatial-dependent capacitance *C*_*c*_(*z*). (**b**) Relative value of the spontaneous emission rate as a function of the normalized coupling capacitance, *c* = *C*_*c*_/*C*_*J*_, for a model with (solid) and without (dashed) **A**^2^ term. We assume *C*_*J*_ = 25fF, *Z*_0_ = 50Ω and a qubit gap *ω*_0_ = 2*π* × 7.5 GHz. Note that *c* = 0 corresponds to infinite separation between the qubit and the line, as *c* ∝ 1/*z*.

The interplay of both effects is now studied with a particlular physical system, a transmon that is capacitively coupled to an open transmission line, as depicted in Fig. 1, while at a finite height *z* above the waveguide. The equivalent circuit to this setup, shown in Fig. 3(a), is similar to the one for an in-plane transmon^42^. From an analysis of such effective model, we obtain expressions for the diamagnetic term and for the dipolar coupling (see Methods for a more detailed derivation)


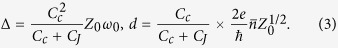


Here *ω*_0_ is the qubit frequency, *Z*_0_ is the line impedance, *C*_*J*_ is the transmon’s capacitance, *C*_*c*_ is the qubit-line coupling capacitance and 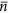 is the matrix element of the number operator on the lowest transmon energy levels.

From these expressions it follows that *J*(*ω*) = 2*πd*^2^*α*(Δ)*ω* cannot have a monotonic behavior with respect to the coupling strength: while *d*^2^ grows as 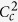, *α* decreases with 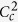 and the product of both must saturate or decrease at large couplings. To analyze this behavior we introduce the relative capacitance *c* = *C*_*c*_/*C*_*J*_, with which we can express the evolution of the spontaneous emission rate of the qubit onto the line as


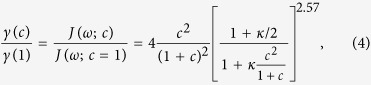


where *κ* = 6.77*C*_*J*_*Z*_0_*ω*_0_. This expression has to be compared with the one that we would obtain without **A**^2^ renormalization, which would be


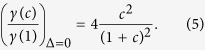


Figure 3(b) shows the behavior of both functions for a transmon qubit with *C*_*J*_ = 25fF, *Z*_0_ = 50Ω and *ω*_0_ = 2*π* × 7.5 GHz. Due to the weak anharmonicity, such a qubit *is not in the ultrastrong coupling regime* and is restricted to coupling strengths 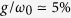. Nevertheless, even then we show evidence of non-monotonic behavior of the spontaneous emission rate as described by *γ*(*c*).

In order to experimentally probe this behavior we would need a qubit with a tunable coupling capacitance. Such a setup already exists: it consists of a mobile transmon that is suspended at a height *z* on top on the transmission line [cf. Fig. 1], as in the experiment by Shanks *et al*.^39^. Based on our theoretical study, the solid line in Fig. 3(b) suggests that, when the transmon is initially far away and *c* ≃ 0, the spontaneous emission would be small, due to an effective decoupling of both elements. However, when the transmon approaches the line, *c* grows as 1/*z*, and the spontaneous emission rate increases until it saturates and starts decreasing again due to the effective decoupling mentioned before. By probing different separations between the transmon an a transmission line, and measuring how much energy the qubit deposits onto the transmission line when spontaneously decaying, one should see a dependence similar to Fig. 3(b), where *c* ∝ 1/*z*. This analysis does not consider the coupling to out-of-plane electromagnetic modes as the qubit rises: these decay channels add up to the total emission rate of the qubit, but do not affect the emission into the line and have a negligible contribution of **A**^2^ terms.

## Discussion

Our study has shown that the **A**^2^ term decreases the effective light-matter coupling in a superconducting circuit. However, this decrease cannot be observed in a traditional experiment with superconducting qubits because, even with the diamagnetic term, the effective model remains Ohmic and the only measurable parameter, the interaction strength *α*, combines the strengths of the dipolar and diamagnetic terms in an inseparable way.

Inspired by this problem, we have suggested a setup in which not only the effective decoupling can be measured, but where the actual model for light-matter interaction in a superconducting circuit can be asserted. This setup is a transmon that is suspended on top of a transmission line. We have shown that the same effective circuit parameters that allow increasing the dipolar coupling strength, *d*, also cause a growth of the **A**^2^ term. This has the consequence that the actual coupling strength will eventually saturate and decrease as a function of the transmon-line separation, allowing an independent calibration of Δ and *d*. Most important, this non-monotonic behavior occurs for all ranges of the interaction, not only in the USC/DSC regimes, making a transmon a suitable qubit for such experiments.

The implementation of an experiment such as the one suggested in this work would represent the first experimental evidence of the light-matter decoupling effect, as initially predicted for linear systems^25^ here extended to individual few-level systems. Moreover, it would also represent the first evidence and calibration of the **A**^2^ term in circuit quantum electrodynamics. Such measurements would be extremely relevant for studying the influence of this term in the superradiant phase transition^29^, or in the study of qubit-qubit interactions^43^, where it could lead to additional deviations from the traditional theory^44^.

## Methods

### Interpolation of the spectral function

We now summarize the methods that lead to the previous results. We start adding to Eq. 1 an expression for the field weights *f*_*k*_, based on a model for a microwave guide as a chain of coupled oscillators, 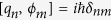, with cutoff frequency *ω*_*c*_


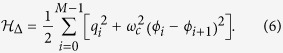


For periodic boundary conditions, the lattice is diagonalized by plane waves with quasimomenta 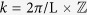, where L = *Mδx* is the actual resonator size and *δx* is the spacing of the discretisation. The lattice dispersion is approximately linear around the qubit, *ω*_*k*_ = *v*|*k*|, with 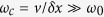 and a speed that we use to define a length scale, *v* = 1. The field operators now read


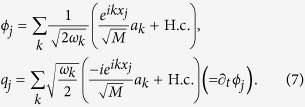


While *ϕ* and *q* are related to the flux and charge operators in circuit-QED, they lack microscopic parameters (inductances, capacitances, etc) that are abstracted into the dipole moment *d* and quadratic weight Δ.

We study two *F* operators, reproducing the transmon’s capacitive coupling to the voltage of the transmission line, *F*^(*cq*)^, and the flux qubit’s inductive coupling to the intensity running through the line, *F*^(*fq*)^


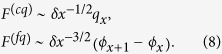


These expressions ensure that the spectral function is independent of the discretization at low energies^45^


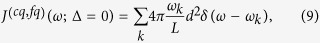


and leads to the expected Ohmic behavior.

Let us regard the effect of the *F*^2^ term. We recast the resonator Hamiltonian in the matrix form 
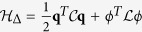
, and diagonalize it with a canonical transformation. The new eigenmodes and eigenfrequencies are used to reexpress the *F* operator and to compute the new spectral function, *J*(*ω*; Δ). In order to do this, we first fix a length L = 10*λ*_0_ = 20*πv*/*ω*_0_ that ensures a small level spacing d*ω* = 2*πv*/L*ω*_0_ ≪ 1. We then diagonalize the problem for increasingly finer discretizations, *δx*, doing a finite size scaling to obtain the pairs of frequencies and couplings, {*ω*_*n*_(Δ), *f*_*n*_(Δ)}, in the limit *δx* → 0. Using these values we compute a function


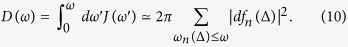


*D*(*ω*) is fitted and then differentiated to obtain *J*(*ω*), working carefully with the interpolation of *D*(*ω*) to eliminate finite size and discretization effects that do not contribute in the continuum limit.

**Figure 4:**
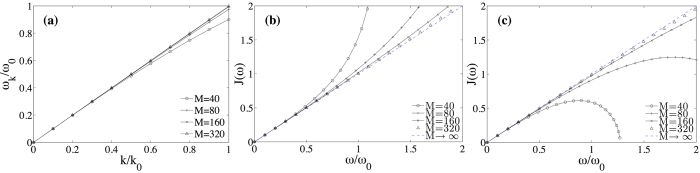
Spectral properties without the A^2^ term (Δ = 0), as a function of the normalized frequencies *ω*_*k*_ = *ω*_*k*_/*ω*_0_ and momenta. Mode eigenfrequencies (**a**) and spectral functions for the capacitive (**b**) and inductive coupling (**c**). The simulation assumes a waveguide with *L* = 10*λ*_0_, where *λ*_0_ is the wavelength associated to *ω*_0_, and uses *M* = 40,80,160 and 320 modes to extrapolate the dispersion relation.

To exemplify such a procedure and test its convergence, in Fig. 4 we show the results for the linear case (Δ = 0), in which we know that the bath is Ohmic and we have thus to recover a linear density. Note the convergence to a linear dispersion relation, 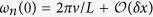, already for 60 modes. The spectral function rapidly becomes linear, 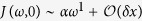, allowing us to extrapolate the coefficient *α* in the limit *δx* → 0.

### Effective circuit of the transmon in a line

Finally, for completeness, we discuss the effective circuit Hamiltonian, *H* = *H*_*qb*_ + *H*_*int*_ + *H*_Δ_, that we have used to discuss the transmon-line coupling:


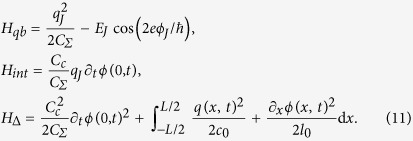


The first line provides the qubit eigenenergies, 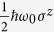, built from the canonical variables of the transmon: the flux *ϕ*_*J*_ and the charge *q*_*J*_. The second line is the capacitive coupling between the qubit and the line, *dσ*^*x*^*F*, which is a function of the coupling and total capacitances, *C*_*c*_ and *C*_Σ_ = *C*_*c*_ + *C*_*J*_. Finally, the third term contains the line Hamiltonian for its charge and flux distributions, *q*(*x*, *t*) and *ϕ*(*x*, *t*), renormalized by the quadratic term that arises from the qubit-line coupling. The capacitance and inductance per unit length determine the speed of light *v* = (*c*_0_*l*_0_)^−1/2^ and also appear in the particular expression of the coupling operator


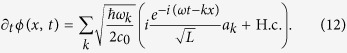


Substituting the expression for the field and relating it to the model that we solved numerically before, we obtain


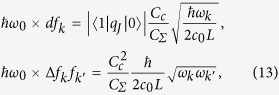


where the charge operator is evaluated between two eigenstates of the qubit. This leads to the relation


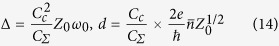


where 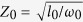 is the impedance of the line and 
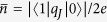
 is the matrix element of the number operator between two lowest transmon energy levels.

## Additional Information

**How to cite this article**: García-Ripoll, J. J. *et al*. Light-matter decoupling and A^2^ term detection in superconducting circuits. *Sci. Rep*. **5**, 16055; doi: 10.1038/srep16055 (2015).
