# Corrigendum: Light-matter decoupling and **A**^2^ term detection in superconducting circuits

**DOI:** 10.1038/srep40470

**Published:** 2017-01-19

**Authors:** J. J. García-Ripoll, B. Peropadre, S. De Liberato

Scientific Reports 5: Article number: 16055; 10.1038/srep16055 published online: 11 04 2015; updated: 01 19 2017.

The original version of this Article contained a typographical error in Reference 24, which was incorrectly given as:

T. Tufarelli, K. R. McEnery, S. A. Maier & M. S. Kim. Phys. Rev. A 89, 023817 (2014).

This has been corrected in the HTML and PDF versions of this Article.

